# Abdominal irradiation modulates 5-Fluorouracil pharmacokinetics

**DOI:** 10.1186/1479-5876-8-29

**Published:** 2010-03-25

**Authors:** Chen-Hsi Hsieh, Yen-Ju Hsieh, Chia-Yuan Liu, Hung-Chi Tai, Yu-Chuen Huang, Pei-Wei Shueng, Le-Jung Wu, Li-Ying Wang, Tung-Hu Tsai, Yu-Jen Chen

**Affiliations:** 1 Institute of Traditional Medicine, School of Medicine, National Yang-Ming University, Taipei, Taiwan; 2 Department of Radiation Oncology, Far Eastern Memorial Hospital, Taipei, Taiwan; 3 Department of Radiation Oncology, Mackay Memorial Hospital, Taipei, Taiwan; 4 Department of Gastrointestinal Division, Mackay Memorial Hospital, Taipei, Taiwan; 5 Department of Medical Research, Mackay Memorial Hospital, Taipei, Taiwan; 6 Department of Education and Research, Taipei City Hospital, Taipei, Taiwan; 7 Genetics Center, Department of Medical Research, China Medical University Hospital, Taichung, Taiwan; 8 Graduate Institute of Chinese Medical Science, China Medical University, Taichung, Taiwan; 9 Department of Radiation Oncology, National Defense Medical Center, Taipei, Taiwan; 10 School and Graduate Institute of Physical Therapy, College of Medicine, National Taiwan University, Taipei, Taiwan

## Abstract

**Background:**

Concurrent chemoradiation with 5-fluorouracil (5-FU) is widely accepted for treatment of abdominal malignancy. Nonetheless, the interactions between radiation and 5-FU remain unclear. We evaluated the influence of abdominal irradiation on the pharmacokinetics of 5-FU in rats.

**Methods:**

The radiation dose distributions of cholangiocarcinoma patients were determined for the low dose areas, which are generously deposited around the intrahepatic target volume. Then, corresponding single-fraction radiation was delivered to the whole abdomen of Sprague-Dawley rats from a linear accelerator after computerized tomography-based planning. 5-FU at 100 mg/kg was intravenously infused 24 hours after radiation. A high-performance liquid chromatography system equipped with a UV detector was used to measure 5-FU in the blood. Ultrafiltration was used to measure protein-unbound 5-FU.

**Results:**

Radiation at 2 Gy, simulating the daily human treatment dose, reduced the area under the plasma concentration vs. time curve (AUC) of 5-FU by 31.7% compared to non-irradiated controls. This was accompanied by a reduction in mean residence time and incremental total plasma clearance values, and volume of distribution at steady state. Intriguingly, low dose radiation at 0.5 Gy, representing a dose deposited in the generous, off-target area in clinical practice, resulted in a similar pharmacokinetic profile, with a 21.4% reduction in the AUC. This effect was independent of protein binding capacity.

**Conclusions:**

Abdominal irradiation appears to significantly modulate the systemic pharmacokinetics of 5-FU at both the dose level for target treatment and off-target areas. This unexpected and unwanted influence is worthy of further investigation and might need to be considered in clinical practice.

## Background

Concurrent use of chemotherapy and radiation therapy (CCRT) is becoming the standard treatment for various malignancies, especially locally advanced cancers. 5-Fluorouracil (5-FU) is one of the most commonly used and classical chemotherapeutic agents of CCRT. It is used as a neoadjuvant, definitive, or adjuvant treatment for cancers arising from the esophagus [1], biliary tract [2], pancreas [3], stomach [4], rectum [5], and bladder [6], in combination with RT.

Pharmacokinetics is the study of a drug and/or its metabolite kinetics in the body and what the body does to the drugs [7]. Pharmacokinetic properties of drugs are affected by elements such as the site of administration and the concentration at which the drug is administered. Modulation of pharmacokinetics of anti-cancer drugs, such as 5-FU, is reportedly influential on disease-free survival (DFS) rates for colorectal cancer [8].

Three-dimensional conformal radiotherapy (3DCRT), intensity-modulated radiotherapy (IMRT), and tomotherapy are currently used for cancer treatment worldwide. These therapies are supposed to produce greater target dose conformity and better critical organ sparing effects, allowing target dose escalation, with lower toxicity to normal tissues [9-12]. Nonetheless, each is usually accompanied by general, low-dose distribution to the torso. Yet, no comprehensive understanding regarding the biological effects of this general, low-dose distribution is established.

With abdominal RT, including intent-to-treat hepatic lesions, it is usually inevitable to irradiate part of the liver, the largest organ occupying at least one third of the upper abdomen. Since the liver is the major site of metabolism for the majority of chemotherapeutic agents, it is rational to hypothesize that RT could influence the pharmacokinetics of anti-cancer drugs. However, no data regarding to the interaction of RT and pharmacokinetics is published. In the present study, we investigated the effect of RT, including therapeutic fraction size and off-target dose, on the pharmacokinetics of 5-FU in rats. The conceptual correlation to clinical practice in humans is drawn from point of view of the radiation oncologist.

## Materials and methods

### Treatment planning selection

Prior to the pharmacokinetic analysis in rats, we demonstrated the concept that low dose radiation distribution areas are generously deposited around the intrahepatic target volume in cholangiocarcinoma patients. From 1 January 2008 through 30 September 2008, treatment plans of four cholangiocarcinoma patients receiving CCRT were retrospectively reviewed and various treatment planning results were compared. Approval for the study was obtained from the Institutional Review Board of Far Eastern Memorial Hospital. All patients had American Joint Committee on Cancer Stage IIIA.

### Target and treatment planning

Although patients were treated by only one mode of RT, four sets of radiation plans were made for each patient including that for conventional radiotherapy (2DRT), 3DCRT, IMRT, and tomotherapy. The PINNACLE^3 ^version 7.6c planning system for the former three modes and the Hi Art Planning system for tomotherapy (Tomotherapy, Inc., Madison, Wisconsin, USA) were used. Normal liver was defined as the total liver volume minus the gross tumor volume. The treatment fields for 2DRT, 3DCRT, and IMRT were 2, 4, and 7, respectively. The field width, pitch, and modulation factor (MF) used in tomotherapy were 2.5 cm, 0.32, and 3.5, respectively. A fraction size of 2 Gy was chosen as the daily dose. For the radiation dose to the normal liver, an isodose line of 0.5 Gy was designed to represent the off-target, general low-dose area during daily treatment.

### Materials and reagents

The 5-FU and high-performance liquid chromatography (HPLC)-grade methanol were purchased from Sigma Chemicals (St. Louis, MO, USA) and Tedia Company, Inc. (Fairfield, OH, USA), respectively. Milli-Q grade (Millipore, Bedford, MA, USA) water was used for the preparation of solutions and mobile phases.

### Animals and sample preparation

Adult, male Sprague-Dawley rats (300 ± 20 g body weight) were provided by the Laboratory Animal Center at National Yang-Ming University (Taipei, Taiwan). They were housed in a specific pathogen-free environment and had free access to food (Laboratory Rodent Diet 5001, PMI Nutrition International LLC, MO, USA) and water. All experimental animal surgery procedures were reviewed and approved by the animal ethics committee of Mackay Memorial Hospital, Taipei, Taiwan (MMH-A-S-98011).

The rats were anesthetized with urethane 1 g/ml and α-chloralose 0.1 g/ml (1 ml/kg, intraperitoneal injection), and were immobilized on a board to undergo computed tomography for simulation of the whole abdominal field. The cranial margin was set at 5 mm above the diaphragm. 2DRT was used to deliver the radiation dose. The experimental animals were randomized to control (0 Gy), 0.5, and 2 Gy groups. Each group's data was collected from 6 to 8 rats per group (6 for controls, 8 for 0.5 Gy, and 7 for 2 Gy).

Allometric scaling of the radiation doses (0.5 and 2 Gy) between humans and rats, respectively, was an important consideration in this study. In a literature review, we found no direct comparison of allometric scaling using abdominal irradiation. Thus, we compared the scaling data from total-body irradiation of rats and humans instead. The lethal dose (LD50) is defined as the dose of any agent or material that causes a mortality rate of 50% in an experimental group within a specified period of time. The allometric scaling of LD50 (Gy) of total-body irradiation for human and rat is 4 Gy and 6.75 Gy, respectively [13]. Given that this difference is moderate, we decided to use 0.5 and 2 Gy for rats to simulate the relevant dose range for daily treatment of human torso.

Ambre et al. [14] studied the elimination of 5-FU and its metabolites after intravenous administration of 5-FU at 15 and 150 mg/kg to rats. The results of that study suggested that saturation of the catabolic pathway occurred after the higher dose. Jarugula et al. [15] proved that the dose-normalized area under the curve (AUC) was significantly higher after administration of 100 mg/kg (mean ± standard deviation, SD, 1.14 ± 0.55 mg· h/L/mg) than after 50 mg/kg (mean ± SD, 0.50 ± 0.16 mg· h/L/mg) or 10 mg/kg (mean ± SD, 0.43 ± 0.11 mg· h/L/mg). Based on these studies, we chose 100 mg/kg as a feasible 5-FU dose in rats for examination of 5-FU pharmacokinetic parameters.

Twenty hours after RT, the rats were administered 100 mg/kg 5-FU in 2 mL of normal saline by intravenous infusion into the femoral vein over a 2-min period [15]. A 150-μL blood sample was withdrawn from the jugular vein with a fraction collector according to a programmed schedule at 5, 15, 30, 45, and 60 min, and 1.5, 2, 2.5, and 3 h following drug administration. The blood samples were immediately centrifuged at 3300 × *g *for 10 min. The resulting plasma (50 μL) was added to 1 mL of ethyl acetate a clean tube, vortexed for 5 min, and centrifuged at 5900 × *g *for 10 min. After centrifugation, the upper organic layer containing the ethyl acetate was transferred to a new tube and evaporated to dryness under flowing nitrogen. The dried residue was reconstituted with 50 μL of Milli-Q water (Millipore). A 20-μL aliquot of the solution was injected to the high performance liquid chromatography-ultraviolet (HPLC-UV) detection system.

### Liquid chromatography

Chromatographic analysis was performed on a Model LC-20AT HPLC system (Shimadzu, Tokyo, Japan) equipped with a Model SPD-20A wavelength UV detector, SIL-20AC autosampler, and an LC Solution data processing system. A LiChroCART RP-18e column (Purospher, 250 mm, 5 μm, Merck, Darmstadt, Germany) with a LiChroCART 4-4 guard column was used for separation. The mobile phase comprised 10 μM potassium phosphate-methanol (99: 1, v/v, pH 4.5 adjusted by 85% phosphoric acid), and the flow rate of the mobile phase was 1 ml/min. The detection wavelength was set at 266 nm.

### Protein binding

The protein binding of 5-FU was determined by ultrafiltration. The 150 μL of plasma was divided into two parts; 50 μL of plasma was used to measure the total concentration of 5-FU, while the remaining plasma was transferred to an ultrafiltration tube (Centrifugal, Millipore, Bedford, MA, USA) for measurement of free 5-FU.

### Pharmacokinetics and data analysis

Pharmacokinetic parameters such as the AUC for concentration vs. time, terminal elimination phase half-life (t_1/2_), maximum observed plasma concentration (Cmax), mean residence time (MRT), total plasma clearance (CL), volume of distribution at steady state (Vss), and the elimination constant (Kel) were calculated by the pharmacokinetics calculation software WinNonlin Standard Edition, Version 1.1 (Scientific Consulting, Apex, NC, USA) using a compartmental method.

### Statistical methods

The results are presented as means ± standard deviations. Differences in actuarial outcomes between the groups were calculated using one-way analysis of variance (ANOVA), with post hoc multiple comparisons. All analyses were performed using the Statistical Package for the Social Sciences, version 12.0 (SPSS, Chicago, IL, USA).

## Results

### Comparison of treatment plans for different radiation dosing techniques

**Figure 1:**
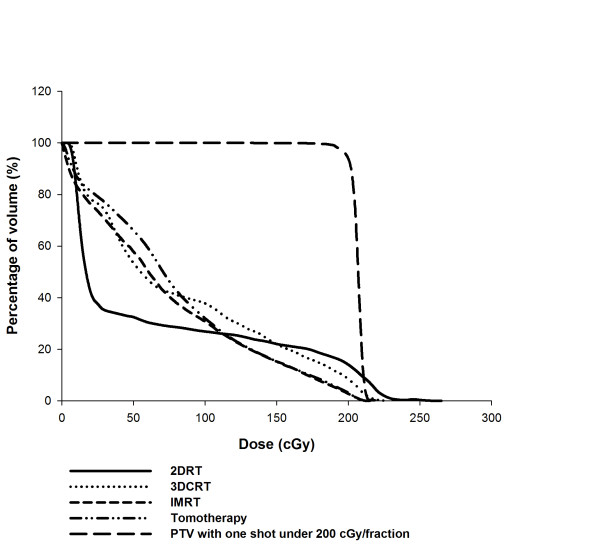
**The dose-volume histogram of the normal liver under different modalities**. The average dose-volume curve of the normal liver under different modalities with 2 Gy to the tumor bed using the dose-volume histogram evaluation for the four patients. The transverse axis illustrates delivered dose in cGy and the vertical axis represents the percentage of liver's volume.

**Figure 2:**
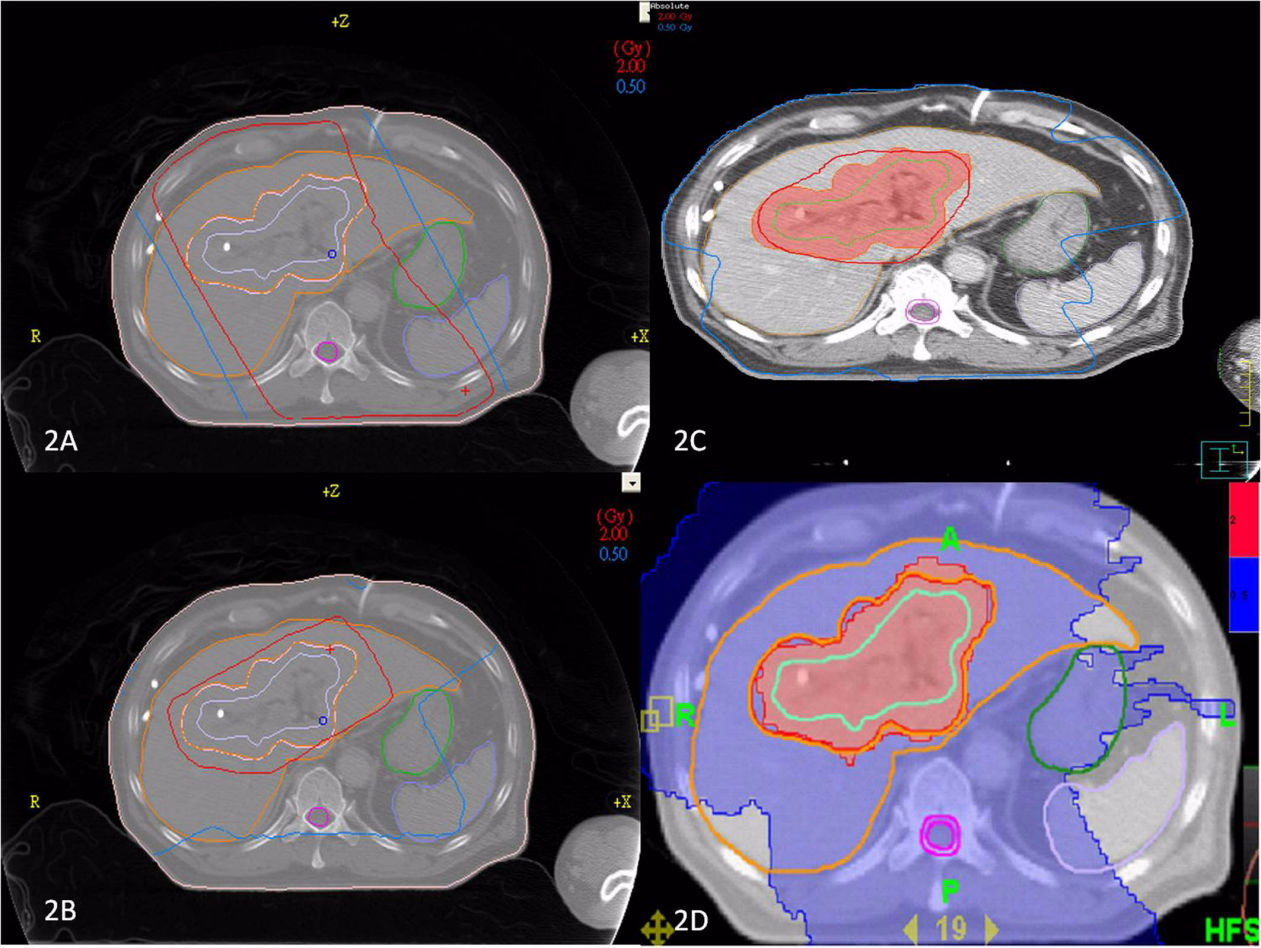
**Isodose distribution by different irradiation techniques**. An example of isodose distribution using different irradiation techniques delivering 2 Gy to the tumor bed for one cholangiocarcinoma patient. A) The conventional radiation therapy (2DRT). B) Three-dimensional conformal radiotherapy (3DCRT). C) Intensive modulated radiotherapy (IMRT). D) Tomotherapy. Orange line, liver; green line, stomach; bright orange line, planning target volume; purple line, clinical target volume for 2DRT and 3DCRT; light green line, IMRT and tomotherapy. The areas for 2 Gy and 0.5 Gy were contoured with red and blue color lines for 2DRT, 3DCRT and IMRT, respectively. The areas for 2 Gy and 0.5 Gy are red and blue, respectively, for tomotherapy.

In the clinical setting, the liver volumes of the cholangiocarcinoma patients receiving 0.5 Gy in daily 2 Gy doses were estimated using a dose-volume histogram for 2DRT, 3DCRT, IMRT, and tomotherapy. The mean ± SD of the liver volumes of the four patients was 1394 ± 94 cc. The liver volumes receiving 0.5 Gy were 32.5%, 53.5%, 57.9%, and 66.1%, respectively (Figure 1). A representative example of isodose distribution with 2 Gy to the targets using the different techniques is illustrated in Figure 2. It suggests that the low-dose radiation area generously deposits around the intrahepatic target volume, especially when advanced, conformal radiation techniques are used.

### Chromatographic analysis and method validation

Under the conditions described above, the retention time of 5-FU was 5.4 min. The linearity of calibration curves was demonstrated by the good determination coefficients (*r*^2^) obtained for the regression line. Good linearity was achieved over the range 0.01-5 μg/ml, with all coefficients of correlation greater than 0.998. All samples were freshly prepared, including the standard solutions, from the same stock solution (5 mg/mL). The 0.01-μg/mL limit of quantification was defined the lowest concentration on the calibration curve that could be measured routinely with acceptable bias and relative SD.

The overall mean precision, defined by the relative SD, ranged from 0.2% to 11.0%. Analytical accuracy was expressed as the percentage difference of the mean observed values compared to known concentrations varying from -10.0% to 14.0%. The recovery results for concentrations of 0.1- 10 μg/mL were 92.0%- 94.0%.

### Pharmacokinetics of 5-FU

**Figure 3:**
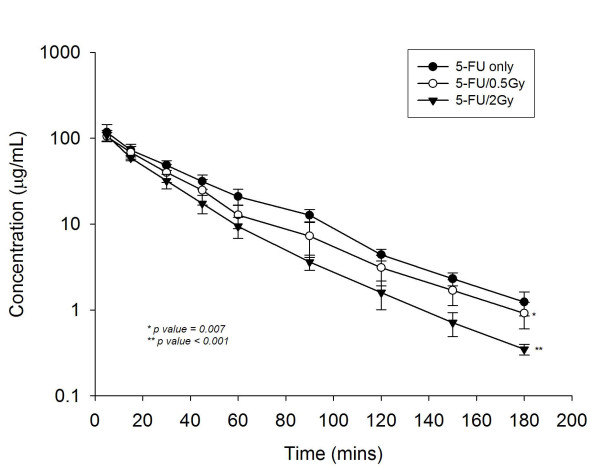
**The area under the curve (AUC) for plasma concentration versus time of 5-FU**. The AUC of 5-FU 100 mg/kg to rats in the control, 0.5-, and 2-Gy groups. The transverse axis illustrates time in minutes and the vertical axis represents the concentration of 5-FU in the plasma.

To verify that local RT modulated the systemic pharmacokinetics of 5-FU, we established an experimental model using CT-based planning and whole abdominal irradiation in rats, and merged it to our pharmacokinetics assay system. Intriguingly, we found that irradiation markedly reduced the AUC of 5-FU in rats by 21.4% at 0.5 Gy (*p *= 0.007) and 31.7% at 2 Gy (*p *< 0.001), respectively (Figure 3). Of special interest, the radiation at 2 Gy to the rat abdomen simulated the daily treatment dose to a human, approximating the low-dose radiation (0.5 Gy) deposited in the generous, off-target area in clinical practice. Irradiation significantly decreased T_1/2 _and MRT (*p *= 0.02 for the 0.5-Gy group and *p *< 0.001 for 2-Gy group), and by contrast, increased the CL (*p *= 0.03 for the 0.5-Gy group and *p *< 0.001 for the 2-Gy group), and Vss (*p *= 0.05 for the 0.5-Gy and for the 2-Gy groups, respectively) of 5-FU when compared to non-irradiated controls (Table 1). There was no significant difference in the values of Cmax and Kel within any group.

**Table 1:** 5-Fluorouracil (100 mg/kg, i.v.) pharmacokinetics in rats after irradiation with and without 0.5 and 2 Gy.

Parameters	Controls	Whole abdomen irradiation	
		
	0 Gy	0.5 Gy	2 Gy
AUC (min μg/mL)	4641 ± 414	3647 ± 726*	3168 ± 270*^†^
t_1/2 _(min)	32.3 ± 10	30.3 ± 2.5	26.9 ± 4.0*
Cmax (μg/mL)	160.0 ± 33	131 ± 19	146 ± 27
MRT (min)	36.0 ± 2.7	31 ± 4.2*	25 ± 1.5*^†^
CL (mL/kg/min)	21.0 ± 1.9	28.5 ± 7.3*	31.7 ± 2.6*^†^
Vss (mL/kg)	798.0 ± 89	885 ± 96*	824 ± 89*
Kelgo1/minp	0.026 ± 0.001	0.031 ± 0.004	0.037 ± 0.001

AUC: area under the plasma concentration *vs*. time curve; t_1/2_: terminal elimination phase half-life; Cmax: maximum observed plasma concentration; MRT: mean residence time; CL: total plasma clearance; Vss: volume of distribution at steady state; Kel: elimination constant.

*The mean difference is significant at the 0.05 level in comparison to the control group.

^†^The mean difference is significant at the 0.05 level between the 0.5 and 2 Gy groups.

### Protein binding

We next examined whether the differences involved protein binding of 5-FU in plasma. Protein binding of 5-FU in rat plasma ranged from 62% to 66% among the different groups. Protein bound/unbound ratios of 5-FU did not differ by radiation dose or post-radiation interval.

## Discussion

Advances in radiation technology have provided better conformal dose distribution to simultaneously hit the target lesions and spare critical organs [9-12]. Nonetheless, areas other than the target area are exposed to significant low dose radiation, making radiation oncologists uncomfortable with this uncertainty in daily practice. Most of this concern comes from a deficiency of knowledge about the biological effects of exposure to radiation within the general, low-dose volumes, especially those exposures produced by the latest advanced technologies. In the clinical cases treated with different techniques, we noted that more than 50% of the normal liver was exposed to 0.5 Gy during daily 2-Gy radiation treatments, except when using 2DRT to treat cholangiocarcinoma patients. In the corresponding animal model, we found, for the first time, after an extensive literature review, that local RT, not only at the therapeutic 2-Gy fraction, but also at 0.5 Gy (representing a dose deposited in the general, off-target area in clinical practice), modulated systemic 5-FU pharmacokinetics. Paolo et al. reported that colorectal cancer patients given radiation doses resulting in lower 5-FU AUC had reportedly lower DFS rates [8]. Thus, the reduction of the 5-FU AUC caused by RT could influence the outcomes of cancer patients receiving abdominal CCRT to an extent that demands our consideration and is not negligible. Therefore, the pharmacokinetics of 5-FU during CCRT should be rechecked and the optimal 5-FU dose should be reevaluated, and adjusted if necessary, during CCRT.

The liver catabolyzes about 80% of 5-FU via the dihydropyrimidine dehydrogenase (DPD) pathway to generate toxic 5-fluoro-5,6-dihydro-uracil (5-FDH2), whereas the anabolic pathway, via orotate phosphoribosyl transferase (OPRT), produces active metabolites including 5-fluorouridine-5'-monophosphate (FUMP), 5-fluorouridine (5-FUrd), and 5-fluoro-2'-deoxyuridine (5-FdUrd) [16,17]. To elucidate which pathway was involved or was affected by RT-induced pharmacokinetic alteration, further assays for the activities of DPD and OPRT are of importance.

It is possible that metabolic and excretory systems dysfunction in such radiation-induced reductions of 5-FU AUC. Since the liver falls into the irradiated volume, DPD, a rate limiting step in the catabolism of 5-FU [18], may be affected by radiation injury to liver. About 80% of the administered 5-FU is degraded by DPD [19]. Because 5-FU has a relatively narrow therapeutic index, a strong correlation is described between exposure to 5-FU and both hematologic and gastrointestinal toxicity [20]. The biochemical basis of severe 5-FU toxicity is attributed to impaired drug catabolism, resulting in a markedly prolonged 5-FU plasma t_1/2 _and almost complete absence of drug catabolites [21]. Additionally, there is ample evidence to suggest that systemic low DPD activity is associated with an increased risk of development of severe 5-FU-associated toxicity. The overall toxicity was twice as high in patients with profound DPD deficiencies (< 45% of the mean DPD activity of a control population) when compared to patients with moderate DPD deficiencies (between 45% and 70% of the mean DPD activity of a control population), as reported by Milano et al. [22]. In addition, mutations and single nucleotide polymorphisms (SNPs) can cause deficiencies in DPD enzymatic activity, and patients with DPD deficiencies have a reduced capacity to metabolize 5-FU and are at risk of developing severe toxic reactions [23-25].

The kidney is another organ located within the irradiated volume in the current study. From 10% to 20% of 5-FU is excreted unchanged in the urine [26]. For patients with renal dysfunction, the plasma concentration of 5-FU on nondialysis days is significantly higher than on dialysis days, and this may be due to the removal of some factors from plasma by hemodialysis, which inhibit DPD activity [27]. Because the therapeutic index for 5-FU is relatively narrow and correlated with hematologic and gastrointestinal toxicity [20], decreased renal function may lead to increased systemic exposure and increased toxicity. Therefore, possible renal dysfunction induced by radiation could have influenced the PK of 5-FU in the current study.

However, the radiation doses used in this study were much less than the tolerable doses to the liver, which in humans is defined as the radiation dose to normal tissue that results in a complication probability of 5% within 5 years after radiotherapy (TD5/5) [28]; the TD5/5 for the human liver is 30 Gy, and for kidneys, it is 23 Gy. The consensus for TD5/5 of liver and kidney in rat is lacking. But the dose could produce detectable hepatic and renal injury has been reported. Whole-liver irradiation of 15-Gy in a single-exposure dose would produce detectable hepatic injury in rats [29] and 25 Gy showed significant histological abnormalities and liver injury, as measured by increased rose bengal retention and liver enzymes [30]. Sharma et al. [31] demonstrated that non lethal doses (10 Gy) cause subtle but immediate changes in renal function and structure in rats. Thus, the possibility that dysfunction of metabolic and excretory systems take place in such radiation-induced reduction of AUC might not be great enough to compromise our findings.

CCRT with 5-FU-based regimens are validated as beneficial for controlling many kinds of cancer, such as those arising from the biliary tract [2], stomach [4], pancreas [3], and rectum [5]. The favorable effects are thought to be mediated through the mechanisms of radiosensitization and combined cytotoxicity and synergy. Our results raise the possibility that RT-modulated 5-FU pharmacokinetics could be one of the mechanisms of action for better tumor control, or for the opposite, for greater complications of CCRT. These possibilities remain to be validated in the clinical setting.

## Conclusions

To our best knowledge, this is the first study proving abdominal irradiation significantly modulates the systemic pharmacokinetics of 5-FU at dosage levels for both the target and off-target areas. For abdominal irradiation with concurrent 5-FU therapy, this unexpected RT-pharmacokinetic influence is worthy of further investigation, which could necessitate reconsideration of 5-FU dosing in clinical practice.

## Competing interests

The authors declare that they have no competing interests.

## Authors' contributions

CH Hsieh participated in the design of the study, performed the radiation and pharmacokinetic experiments, and wrote the manuscript. YJ Hsieh helped CH Hsieh to do some experiments. CY Liu participated in the design of the study. HC Tai was responsible for the radiation planning. YC Huang performed the statistical analysis. PW Shueng collected the clinical data. LJ Wu helped to provide clinical data and information. LY Wang helped to design the experiments. TH Tsai and YJ Chen initiated, organized and supervised all the work, including the manuscript. All authors read and approved the final version of this manuscript.

## References

[B1] Geh JI Bond SJ Bentzen SM Glynne-Jones R Systematic overview of preoperative (neoadjuvant) chemoradiotherapy trials in oesophageal cancer: evidence of a radiation and chemotherapy dose response Radiother Oncol 2006 78 236 244 10.1016/j.radonc.2006.01.009 16545878

[B2] Kim S Kim SW Bang YJ Heo DS Ha SW Role of postoperative radiotherapy in the management of extrahepatic bile duct cancer Int J Radiat Oncol Biol Phys 2002 54 414 419 12243816 10.1016/s0360-3016(02)02952-8

[B3] Moertel CG Frytak S Hahn RG O'Connell MJ Reitemeier RJ Rubin J Schutt AJ Weiland LH Childs DS Holbrook MA Therapy of locally unresectable pancreatic carcinoma: a randomized comparison of high dose (6000 rads) radiation alone, moderate dose radiation (4000 rads + 5-fluorouracil), and high dose radiation + 5-fluorouracil: The Gastrointestinal Tumor Study Group Cancer 1981 48 1705 1710 10.1002/1097-0142(19811015)48:8<1705::AID-CNCR2820480803>3.0.CO;2-4 7284971

[B4] Macdonald JS Smalley SR Benedetti J Hundahl SA Estes NC Stemmermann GN Haller DG Ajani JA Gunderson LL Jessup JM Martenson JA Chemoradiotherapy after surgery compared with surgery alone for adenocarcinoma of the stomach or gastroesophageal junction N Engl J Med 2001 345 725 730 10.1056/NEJMoa010187 11547741

[B5] Krook JE Moertel CG Gunderson LL Wieand HS Collins RT Beart RW Kubista TP Poon MA Meyers WC Mailliard JA Effective surgical adjuvant therapy for high-risk rectal carcinoma N Engl J Med 1991 324 709 715 1997835 10.1056/NEJM199103143241101

[B6] Poortmans PM Richaud P Collette L Ho Goey S Pierart M Hulst M Van Der Bolla M Results of the phase II EORTC 22971 trial evaluating combined accelerated external radiation and chemotherapy with 5FU and cisplatin in patients with muscle invasive transitional cell carcinoma of the bladder Acta Oncol 2008 47 937 940 10.1080/02841860801888799 18568488

[B7] Is there a need for more precise definitions of bioavailability? Conclusions of a consensus workshop, Munich, September 9, 1989; under the patronage of the F.I.P Eur J Clin Pharmacol 1991 40 123 126 10.1007/BF01418409 2065692

[B8] Di Paolo A Lencioni M Amatori F Di Donato S Bocci G Orlandini C Lastella M Federici F Iannopollo M Falcone A 5-fluorouracil pharmacokinetics predicts disease-free survival in patients administered adjuvant chemotherapy for colorectal cancer Clin Cancer Res 2008 14 2749 2755 10.1158/1078-0432.CCR-07-1529 18451241

[B9] Verhey LJ Comparison of three-dimensional conformal radiation therapy and intensity-modulated radiation therapy systems Semin Radiat Oncol 1999 9 78 98 10.1016/S1053-4296(99)80056-3 10196400

[B10] Shueng PW Lin SC Chong NS Lee HY Tien HJ Wu LJ Chen CA Lee JJ Hsieh CH Total marrow irradiation with helical tomotherapy for bone marrow transplantation of multiple myeloma: first experience in Asia Technol Cancer Res Treat 2009 8 29 38 19166240 10.1177/153303460900800105

[B11] Chao KS Low DA Perez CA Purdy JA Intensity-modulated radiation therapy in head and neck cancers: The Mallinckrodt experience Int J Cancer 2000 90 92 103 10.1002/(SICI)1097-0215(20000420)90:2<92::AID-IJC5>3.0.CO;2-9 10814959

[B12] Tai HC Hsieh CH Chao KS Liu SH Leu YS Chang YF Hsiao HT Chang YC Huang DY Chen YJ Comparison of radiotherapy strategies for locally advanced hypopharyngeal cancer after resection and ileocolic flap reconstruction Acta Otolaryngol 2009 129 311 317 10.1080/00016480802163366 18607975

[B13] Vriesendorp HM Van Bekkum DW Broerse JJ, T M Susceptibility to total-body irradiaiton Response to Total-Body Irradiation in Different Species 1984 Amsterdam: Martinus Nijhoff

[B14] Ambre JJ Fischer LJ The effect of prednisolone and other factors on the catabolism of 5-fluorouracil in rats J Lab Clin Med 1971 78 343 353 5092856

[B15] Jarugula VR Lam SS Boudinot FD Nonlinear pharmacokinetics of 5-fluorouracil in rats J Pharm Sci 1997 86 756 758 10.1021/js960451a 9188061

[B16] Bocci G Danesi R Di Paolo AD Innocenti F Allegrini G Falcone A Melosi A Battistoni M Barsanti G Conte PF Del Tacca M Comparative pharmacokinetic analysis of 5-fluorouracil and its major metabolite 5-fluoro-5,6-dihydrouracil after conventional and reduced test dose in cancer patients Clin Cancer Res 2000 6 3032 3037 10955781

[B17] Casale F Canaparo R Serpe L Muntoni E Pepa CD Costa M Mairone L Zara GP Fornari G Eandi M Plasma concentrations of 5-fluorouracil and its metabolites in colon cancer patients Pharmacol Res 2004 50 173 179 10.1016/j.phrs.2004.01.006 15177306

[B18] Lu Z Zhang R Diasio RB Dihydropyrimidine dehydrogenase activity in human peripheral blood mononuclear cells and liver: population characteristics, newly identified deficient patients, and clinical implication in 5-fluorouracil chemotherapy Cancer Res 1993 53 5433 5438 8221682

[B19] Heggie GD Sommadossi JP Cross DS Huster WJ Diasio RB Clinical pharmacokinetics of 5-fluorouracil and its metabolites in plasma, urine, and bile Cancer Res 1987 47 2203 2206 3829006

[B20] Gamelin E Boisdron-Celle M Dose monitoring of 5-fluorouracil in patients with colorectal or head and neck cancer--status of the art Crit Rev Oncol Hematol 1999 30 71 79 10.1016/S1040-8428(98)00036-5 10439055

[B21] Diasio RB Lu Z Dihydropyrimidine dehydrogenase activity and fluorouracil chemotherapy J Clin Oncol 1994 12 2239 2242 7964937 10.1200/JCO.1994.12.11.2239

[B22] Milano G Etienne MC Pierrefite V Barberi-Heyob M Deporte-Fety R Renee N Dihydropyrimidine dehydrogenase deficiency and fluorouracil-related toxicity Br J Cancer 1999 79 627 630 10.1038/sj.bjc.6690098 10027340 PMC2362417

[B23] Deeken JF Figg WD Bates SE Sparreboom A Toward individualized treatment: prediction of anticancer drug disposition and toxicity with pharmacogenetics Anticancer Drugs 2007 18 111 126 10.1097/CAD.0b013e3280109411 17159598

[B24] van Kuilenburg AB Dihydropyrimidine dehydrogenase and the efficacy and toxicity of 5-fluorouracil Eur J Cancer 2004 40 939 950 10.1016/j.ejca.2003.12.004 15093568

[B25] Wei X McLeod HL McMurrough J Gonzalez FJ Fernandez-Salguero P Molecular basis of the human dihydropyrimidine dehydrogenase deficiency and 5-fluorouracil toxicity J Clin Invest 1996 98 610 615 10.1172/JCI118830 8698850 PMC507468

[B26] Peters GJ Peckam M, Pinedo HM, Veronesi U Antimetabolites Oxford Textbook of Oncology 1995 London: Oxford University Press 524 552

[B27] Gusella M Rebeschini M Cartei G Ferrazzi E Ferrari M Padrini R Effect of hemodialysis on the metabolic clearance of 5-Fluorouracil in a patient with end-stage renal failure Ther Drug Monit 2005 27 816 818 10.1097/01.ftd.0000183384.89275.f4 16306860

[B28] Emami B Lyman J Brown A Coia L Goitein M Munzenrider JE Shank B Solin LJ Wesson M Tolerance of normal tissue to therapeutic irradiation Int J Radiat Oncol Biol Phys 1991 21 109 122 2032882 10.1016/0360-3016(91)90171-y

[B29] Geraci JP Mariano MS Jackson KL Radiation hepatology of the rat: time-dependent recovery Radiat Res 1993 136 214 221 10.2307/3578613 8248478

[B30] Geraci JP Mariano MS Jackson KL Hepatic radiation injury in the rat Radiat Res 1991 125 65 72 10.2307/3577983 1824725

[B31] Sharma M Halligan BD Wakim BT Savin VJ Cohen EP Moulder JE The Urine Proteome as a Biomarker of Radiation Injury: Submitted to Proteomics- Clinical Applications Special Issue: "Renal and Urinary Proteomics (Thongboonkerd)" Proteomics Clin Appl 2008 2 1065 1086 10.1002/prca.200780153 19746194 PMC2739391

